# The nasal-oral microbiome axis in allergic rhinitis: environmental triggers, microbial dysbiosis, and immune dysregulation

**DOI:** 10.3389/falgy.2026.1799085

**Published:** 2026-04-28

**Authors:** Yuan Li, Yue Sun, Lei Shi, Aiping Wang, Xue Gao, Hui Leng

**Affiliations:** 1 Liaoning University of Traditional Chinese Medicine, Shenyang, China; 2 The Affiliated Hospital of Liaoning University of Traditional Chinese Medicine, Shenyang, China

**Keywords:** allergic rhinitis, dysbiosis, epithelial barrier, nasal microbiome, oral microbiome, probiotics, short-chain fatty acids, Th2 inflammation

## Abstract

Allergic rhinitis (AR) is a common chronic inflammatory disease, which affects about 400 million people around the world. The role of the upper airway microbiota in the development of AR has recently emerged and seems to be an important player in its pathology, but there are still no detailed mechanistic models that incorporate exposure to the environment, dysbiosis of microbes or dysregulated immunity as a whole. In this review we summarize the state of the art about the microbiome nose-mouth connection in AR to understand how environmental stimuli change the microbiota composition as well as how an imbalance can induce allergy-related inflammation. This review follows a narrative approach. Literature was identified through systematic searches of PubMed, Web of Science, and Scopus databases (up to March 2025) using the following key terms and their combinations: “allergic rhinitis’, “nasal microbiome”, “oral microbiome”, “dysbiosis”, “epithelial barrier”, ’short-chain fatty acids’, “Th2 inflammation”, and “probiotics”. Inclusion criteria encompassed original research articles, systematic reviews, and meta-analyses published in English; conference abstracts, case reports, and purely non-human studies were excluded unless they provided mechanistic insights not available from human data. Environmental exposures substantially alter upper airway microbial communities. Air pollutants such as PM₂.₅ and diesel exhaust particles (DEP) damage epithelial tight junction proteins via reactive oxygen species (ROS), increasing nasal permeability. DEP additionally functions as an immune adjuvant by promoting pro-Th2 immune polarization. Antibiotic treatment during early childhood may affect GI tract development by altering resident bacterial populations, being considered as a strong risk factor for developing AR. On the other hand, farm exposure and microbial diversity provide protection by enhancing regulatory T cell induction. AR patients exhibit characteristic nasal dysbiosis, including overgrowth of Staphylococcus aureus and Moraxella catarrhalis alongside depletion of protective commensals such as Dolosigranulum pigrum and Corynebacterium spp. This dysbiosis disrupts the epithelial barrier, triggering alarmin release (TSLP, IL-25, IL-33) and amplifying type 2 inflammation. The oral microbiota also contributes via the oral-nasal-pulmonar*y* axis whereby periodontal pathogens are pro-inflammatory while commensals have immunomodulatory roles. Mechanistically, microbiome-derived metabolites—especially short chain fatty acids and tryptophan derivatives—regulate the immune system via G protein-coupled receptors, histone deacetylase inhibition, and aryl hydrocarbon receptor activation. Dysbiosis promotes Th2 polarization, Treg/Th17 imbalance, and the activation of ILC2s, whereas neuro-immune interactions via TRPV1/TRPA1 enhance neurogenic inflammation. Translation to clinical opportunity: Microbiome based diagnostic biomarker; Probiotic (nasal/oral); Prebiotics; postbiotics, and engineered bacteria. Multi-omics based precision medicine using ML to stratify patient and tailor intervention. In summary, this review offers an insight into the theory of the microbiome-immunology interplay in AR as well as new avenues to consider regarding treatment of this condition through the nasal-oral microbiota axis.

## 1 Introduction

Allergic rhinitis (AR) is among the most prevalent chronic inflammatory disease in the world with an important burden to society as well as the socioeconomic system. Globally around 400 million people are affected by this condition (1). In a systematic review that included 156 distinct definitions for the term “rhinitis” from 184 publications, the pooled median prevalence of AR was found to be 18.1%, which is reported worldwide with a range of 1%–63% and has been on the rise over time (2).

The costs associated with AR are much higher than traditionally perceived. The Swedish TOTALL study was the first one estimating the total cost of AR on a national scale using questionnaires from people living in Sweden, who were between 18 and 65 years old, calculating health care contacts, medications, absenteeism, and presenteeism (3) with a mean annual direct cost of €210.3, indirect cost of €750.8, resulting in an overall price of €961.1 person/year, where presenteeism contributes to 70% - a silent burden which is usually underestimated (3). Given that Sweden has a population of 9.5 million people and an estimated 24% have AR, the overall annual social cost was estimated to be about €1.3 billion (3).

AR rarely occurs in isolation. The prevalence of AR among asthmatic patients reaches 80%–90%, while 10%–40% of rhinitis patients have concomitant asthma, supporting the concept of ‘united airway disease' (4). This comorbidity pattern underscores the clinical importance of understanding upper airway pathophysiology as a continuum.

In recent years many hypotheses have been presented as an explanation for this rise in allergic disease over the last three decades. The original “hygiene hypothesis” was based on the finding that hay fever became less common when there were more older brothers and sisters at home, based upon data from the 1958 British birth cohort (5). It was hypothesized that infections “from unhygienic contact with older siblings” in the first few years of life could have a protective effect on allergic disease, an inverse relationship repeated many times over in Britain and other wealthy nations (5).

The “biodiversity hypothesis” also pointed out the fact that biodiversity loss and climate change secondary to man's activity are related to several adverse health effects (6). Loss of macrodiversity is associated with shrinking microdiversity, which has been related to changes in the native microbiota (6). Decreased diversity and changed composition of the gut- and skin microbiota is associated to different inflammatory diseases, including asthma, allergic diseases, inflammatory bowel disease, and obesity (6). Comparative studies of the Finns with the Russians from the Karelia region provided important epidemiologic evidence: hay fever was virtually absent from the Russian Karelia, with a mere 2% sensitization against birch pollen as opposed to 27% in Finnish Karelia (7). Russian children had higher abundance of genes-microbes associations and connectivity related to a better homeostasis of immunity and lower incidence of allergies (8).

The “disappearing microbiota hypothesis” postulates that the absence of certain bacteria in the ancestral microbiota have modified the environment in which immunological, metabolic, and cognitive development during the first years of life which lead to epidemics of chronic disease (9). Prenatal and early postnatal life are key time windows for immune system development. The diet, environment, and medical care that an infant experiences determines how the intestinal microbiota is established and progresses, which is used to “train” the developing immune system (10). Between birth and one year old, the immune system “trains” itself as to what antigens it should tolerate (10). Direct evidence was also provided by the CHILD birth cohort study, which showed that children who were diagnosed with allergic disease at age 5 years had a delay in gut microbiota maturity at age 1 year, and that this lag was common across pediatric allergic diseases (atopic dermatitis, asthma, food allergy, and allergic rhinitis) (11).

Collectively, the hygiene, biodiversity, and disappearing microbiota hypotheses converge on a shared premise: insufficient microbial exposure during critical early-life windows disrupts immune homeostasis and predisposes individuals to allergic disease. While existing research has focused predominantly on the gut microbiome, the upper airway—particularly the nasal and oral cavities—represents the primary interface between the external environment and the host immune system. The nasal mucosa is directly exposed to inhaled allergens and pollutants, while the oral cavity is anatomically continuous with the nasopharynx and lower airways through microaspiration routes. Together, these compartments form the first line of host–microbe interaction and constitute a critical node through which environmental signals are transduced into immune responses. Focusing on the nasal-oral microbiome axis therefore represents a natural and necessary extension of these broader theories: it moves the field from general ecological principles toward the organ-specific microbial and immunological mechanisms that directly underlie AR pathogenesis. Moreover, recent evidence demonstrating distinct bacteriome compositions in both the nasal and oral cavities of AR patients compared with healthy controls provides direct microbiological justification for examining this axis as a mechanistic unit rather than studying each compartment in isolation.

Although the relationship between gut microbiome and allergic diseases has been extensively studied, the role of nasal microbiome in AR pathogenesis has only recently begun to receive attention. Host-microbial commensalism can shape innate immune responses in respiratory mucosa, and the nasal microbiome modulates front-line immune mechanisms (13). Analysis of 16S rRNA gene sequences from middle turbinate mucosa samples revealed that AR patients showed significantly distinctive colonization patterns of Firmicutes, Actinobacteria, and Proteobacteria phyla compared to healthy participants (12). The Staphylococcus genus was relatively more abundant in AR patients' nasal mucus, with Staphylococcus aureus-characterized dysbiosis present in AR patients' nasal mucosa (12). A systematic review indicated that Acinetobacter, Corynebacterium, Moraxella, Staphylococcus, and Streptococcus are the dominant genera in children's nasal cavity and nasopharynx (13).

Although great strides have been made in our understanding of the role of microbiome in allergic disease, there are still many important questions remaining unanswered. First, the causality of the link between environmental exposure change and nasal microbiome change is not completely clear; second, the exact immune mechanisms through which nasal microbiome dysbiosis affects the development of AR need to be elucidated. This review aims to systematically integrate research evidence on environmental exposure, nasal microbiome alterations, and AR pathogenesis, focusing on how microbial exposures impact early immune development that could contribute to allergic susceptibility, and investigating possible clinical applications of microbiome-targeted interventions.

## 2 Environmental factors as extrinsic modulators of allergic rhinitis

### 2.1 Air pollution

**Figure 1:**
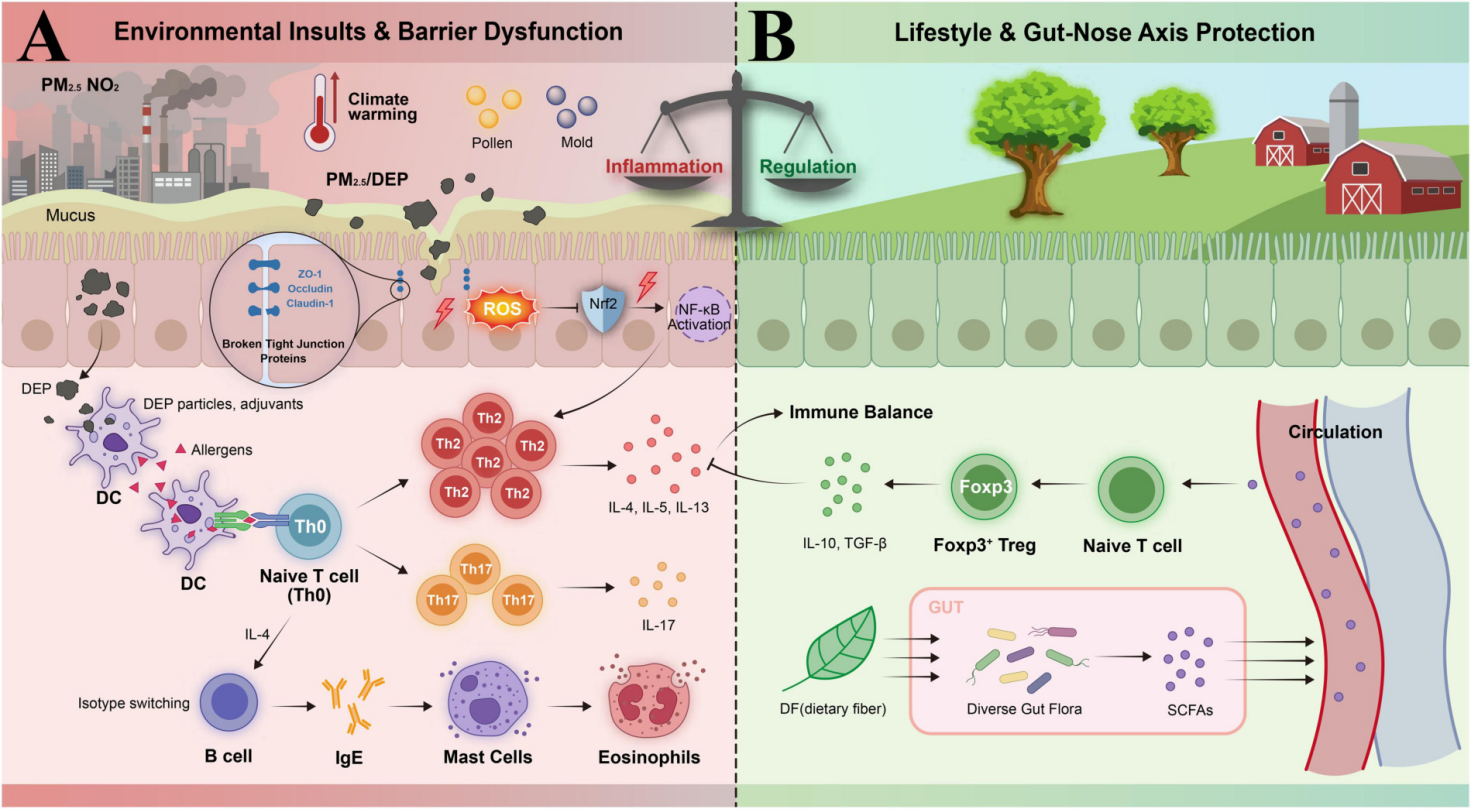
Environmental and lifestyle factors in allergic rhinitis pathogenesis. **(A)** Mechanisms by which air pollution and climate change contribute to disease. Three routes are shown in which air pollutants (PM₂.₅, DEP) and airborne allergens act upon the nasal mucosa: (1) PM₂.₅ promotes ROS production, suppresses Nrf2, up-regulates NF-*κ*B, down-regulates ZO-1, Occludin, Claudin-1; (2) DEP increases DCs antigen presentation and promotes Th2/Th17 differentiation; (3) High levels of Th2 cytokines (IL-4, IL-5, IL-13) and IL-17 promote the generation of IgE and allergic inflammation. **(B)** Lifestyle- and microbiome-mediated protections. Farm life and fibre provide protection. Gut commensals (Clostridium clusters IV/XIVa) ferment fibre into SCFAs, which block HDAC activity, and facilitate the development of Tregs by expressing Foxp3. Exposure to antibiotics at an early age interferes with this route and predisposes to AR.

As illustrated in Figure 1, air pollution has emerged as a significant environmental factor influencing the development and exacerbation of allergic rhinitis (AR). A systematic umbrella review classified PM₂.₅ and NO₂ as suggestive evidence (Class III) environmental risk factors for AR (14).

#### 2.1.1 Epithelial barrier damage by fine particulate matter (PM₂.₅)

PM₂.₅ damages the nasal epithelial barrier by several mechanisms. In a human nasal epithelial cell air liquid interface *in vitro* model, exposure to 50–100 µg/mL PM₂.₅ for 72 h significantly reduced the TER and enhanced the FITC-dextran permeability (15), which was accompanied with the significant down-regulated claudin-1 mRNA and protein expression, and decreased occludin and ZO-1 protein expression (15). Xian et al. also reported significant increases in pro-inflammatory cytokines including IL-8, TIMP-1, and TSLP in the same model (15).

The same results have been reported for the RPMI 2650 human nasal epithelial cell line, where PM₂.₅ exposure induced downregulation of ZO-1, occludin, and claudin-1, reduced the TER, and increased permeability, which was mediated through reactive oxygen species (ROS), as N-acetylcysteine (NAC) pre-treatment mitigated PM₂.₅-induced ROS production and inhibited barrier disruption (16).

Tight junctions consist of transmembrane proteins (occludin, claudin family, JAMs) and intracellular adaptor proteins (ZO-1/2/3). These structures form the critical barrier of nasal epithelium (17). Diesel exhaust particles (DEP) and PM₂.₅ exposure both significantly reduced expression of OCLN, ZO-1, and CLDN1 in primary nasal epithelial cells. Transepithelial electrical resistance (TER, a direct measure of paracellular barrier integrity) decreased, and FITC-dextran permeability increased accordingly, confirming barrier disruption. ROS mediated these effects (17).

#### 2.1.2 Immune adjuvant effects of diesel exhaust particles (DEP)

As one of the main components of urban air pollution, DEP has shown high levels of immune adjuvant activity. Unlike PM₂.₅, DEP was not independently classified as a separate exposure category in the umbrella review (15); its risk contribution is subsumed within the broader fine particulate matter evidence base. The mechanistic immune adjuvant evidence for DEP summarized below therefore derives primarily from experimental and challenge studies rather than meta-analytic epidemiological data. One milestone study demonstrated the adjuvant effect of DEP on human mucosa (18). Of the 15 atopic patients that underwent DEP pretreatment and subsequent nasal immunization with the neoantigen keyhole limpet hemocyanin (KLH), 9/15 (60%) produced KLH-specific IgE antibody. IL-4 was increased in nasal lavage fluid; IFN-*γ* was not changed. In comparison, none of the KLH-alone groups had generated specific IgE (18). This study therefore firstly showed that DEP could function as a mucosal adjuvant for promoting *de novo* IgE sensitisation to neoantigens in human beings.

Combined DEP and ragweed allergen challenge markedly enhanced allergen-specific IgE production (19). Th1-type cytokines (IFN-*γ*, IL-2) were downregulated, while Th2-type cytokines (IL-4, IL-5, IL-6, IL-10, IL-13) were upregulated (19). DEP affects nasal epithelium through three main mechanisms: directing cytokine gene expression toward a Th2 profile, enhancing local antigen-specific IgE production, and driving *in vivo* isotype switching to IgE (20).

#### 2.1.3 Oxidative stress and signaling pathways

PM₂.₅-induced oxidative stress involves multiple signaling pathways. In an OVA-induced AR mouse model combined with PM₂.₅ exposure, the Nrf2 pathway was inhibited (21). PM₂.₅ exposure induced oxidative stress through increased malondialdehyde (MDA) production. Nrf2 signaling pathway activity was inhibited. Antioxidant enzymes SOD and HO-1 expression were reduced. The NF-*κ*B signaling pathway was activated (21). In a combined allergic rhinitis and asthma syndrome (CARAS) mouse model, PM₂.₅ regulated Th1/Th2/Th17 cytokine production through the NF-*κ*B signaling pathway (22). Th2 and Th17 cytokines (IL-4, IL-5, IL-13, IL-17) increased, while Th1 cytokines (IL-12, IFN-*γ*) decreased (22).

### 2.2 Climate change and allergen exposure

#### 2.2.1 Pollen season changes

Climate change also alters the start and duration of the pollen season: pollen emission models coupled to future climate data predict earlier spring pollen emissions for the temperature increase scenario (23) while annual total pollen emissions are predicted to increase significantly as a result of both phenological shifts and temperature-induced increases in pollen production. Considering the promoting effect of the doubling of atmospheric CO₂ concentrations on pollen emission, pollen emissions can increase significantly at the end of the 21st century (23). Trends for longer pollen season length and greater intensity as seen during past decades will aggravate pollen-induced AR and asthma (24).

#### 2.2.2 Indoor allergens

Climate change has an effect on indoor allergens as well, and the systematic umbrella review categorized indoor dampness exposition (OR = 1.49). Indoor dust mite allergen (OR = 1.47, 95%CI: 1.27–1.75) and indoor mold (OR = 1.66, 95%CI: 1.26–2.18) were classified as strong (Class II) environmental risk factors—indicating that residents in damp, mold-contaminated environments face up to 66% higher odds of AR, with clinical implications for indoor environmental control as a preventive strategy (14). A systematic review and meta-analysis of 31 studies found that visible mold was significantly associated with AR (OR = 1.51, 95%CI: 1.39–1.64) and rhinoconjunctivitis (OR = 1.66, 95%CI: 1.27–2.18), with mold odor showing the strongest risk estimates (25). Indoor dampness or mold is consistently associated with AR across diverse populations and study designs, and population-attributable estimates indicate a substantial public health burden (26). Data from eight European birth cohorts further showed that early-life mold or dampness exposure was associated with AR symptoms at school age (aOR=1.12, 95%CI: 1.02–1.23), underscoring the importance of early residential environmental control (27).

### 2.3 Lifestyle and geographic environment

Epidemiological studies show a consistent urban-rural gradient for the prevalence of AR. In the PARSIFAL study, comparison between farm children and references was carried out (28). Farm-reared children had approximately 50% lower odds of rhinoconjunctivitis (aOR=0.50), atopic symptoms (aOR=0.55, 95%CI: 0.42–0.72), and atopic sensitization (aOR=0.53, 95%CI: 0.42–0.67) compared with non-farm controls—effect sizes comparable in magnitude to the risk conferred by early antibiotic exposure, suggesting that microbial diversity from farm environments can substantially offset allergic disease susceptibility (28).

The GABRIEL study followed the subjects prospectively from baseline age 6–11 years to age 20–25 years (29). Living on a farm at both time points was related to lower prevalence of ARs (OR = 0.4, 95%CI: 0.2–0.6). Only baseline farm living was also protective (OR = 0.4, 95%CI: 0.2–0.8) (29). Thus we have confirmation here that the protective effects of farm living against AR continues from childhood through to early adult life and it is in childhood where there is this window of opportunity.

In a population-based cohort of 6,251 adults between the ages of 20 and 44 years who participated in the ECRHS, growing up on a farm during childhood was related to decreased risks of adult atopic sensitization (OR = 0.76, sensitization to cats (OR = 0.63, 95%CI: 0.41–0.96), sensitization to Timothy grass (OR = 0.68, 95%CI: 0.50–0.94) (30).

Some specific protective factors of the farming lifestyle were explored (31). Children from farm families had a substantially lower risk for seasonal AR than children living in rural areas but not on farms (aOR=0.50, 95%CI: 0.33–0.77). Consumption of unpasteurized milk currently had a strong protective effect against atopy (aOR=0.24, 95%CI: 0.10–0.53) regardless of farming status (31) which is consistent with the “hygiene hypothesis”. As Haahtela et al. (6) and Blaser and Falkow (32)have suggested, the progressive loss of diverse early-life microbial exposures may have important consequences for immune development and susceptibility to allergic disease. The association between green space and AR is complicated with inconsistent findings. The biodiversity hypothesis posits that contact with nature increases human microbiome diversity, which fosters a balanced immune system (6). A systematic review and meta-analysis of 21 studies evaluating green space and AR revealed mixed associations (33). The results of the meta-analyses were variable depending on the radius used for buffer zones (33). Prolonged breastfeeding was classified as a Class II protective factor for AR (OR = 0.72, 95%CI: 0.65–0.79) in the umbrella review (14). The mechanistic basis is multifactorial: breast milk provides secretory IgA (sIgA) for passive mucosal protection; human milk oligosaccharides (HMOs) selectively promote *Bifidobacterium* colonization and short-chain fatty acid production, fostering regulatory T cell induction; and milk-derived TGF-*β* and IL-10 directly promote tolerogenic immune programming during the critical neonatal window—all effects that support the establishment of a health-associated early-life microbiome (12, 34).

### 2.4 Antibiotic exposure and Gut microbiome

#### 2.4.1 Early-life antibiotic exposure and AR risk

Early-life antibiotic exposure is closely associated with increased AR risk. The systematic umbrella review classified early antibiotic use as a highly suggestive (Class II) risk factor for AR (OR = 3.73, 95%CI: 3.06–4.55), indicating that children exposed to antibiotics in early life face approximately 3.7-fold higher odds of developing AR compared with unexposed children—representing the strongest modifiable risk factor identified in this umbrella review (14).

In a prospective study on a western Sweden birth cohort, antibiotic exposure during the first week of life was associated with an increase in risk for AR at school age (aOR = 1.75, 95%CI: 1.03–2.97) (35). Preschool farm life lowered risk (aOR=0.31, 95%CI: 0.13–0.78) (18), both of which fit with the hygiene hypothesis. Life-time antibiotic use was also significantly related to AR risk (OR = 2.43, 95%CI: 1.43–4.11) (36).

#### 2.4.2 Gut microbiome dysbiosis and immune mechanisms

Antibiotics increase AR risk by disrupting normal colonization of the gut microbiome. Systematic analysis of gut microbiota composition in AR patients reveals consistent reductions in SCFA-producing genera including *Bifidobacterium*, *Faecalibacterium prausnitzii*, and *Roseburia*, accompanied by decreased fecal butyrate and propionate concentrations, lower peripheral Treg cell frequencies, and elevated circulating Th2 cytokines (IL-4, IL-5, IL-13) relative to healthy controls (37) These interdependent findings are consistent with a mechanistic chain in which gut dysbiosis reduces SCFA availability, impairs Treg induction, and thereby permits unopposed Th2 immune polarization that drives allergic sensitization and AR pathogenesis.

It has been shown how gut Clostridia drive the development of regulatory T cells (Tregs). Spore-forming portion of endogenous intestinal microbiota from mice, especially clusters IV and XIVa of the genus Clostridium, supported colonic Treg cell accumulation (38). Colonisation by a defined mix of Clostridium strains provided a TGF-*β* rich milieu which affected the number and function of colonic Foxp3^+^ Tregs (38). In other studies, 17 strains of Clostridia (cluster IV, XIVa, and XVIII) were also isolated from the human feces (39), which showed high potency for Treg induction. These strains induced Treg expansion and differentiation via provision of bacterial antigen and an abundant supply of TGF-*β* rich milieu (39).

#### 2.4.3 Short-chain fatty acids and immune regulation

The short-chain fatty acids (SCFAs) represent the major microbial fermentation products of dietary fibre, and they are key mediators by which the gut microbiota regulates immunity. SCFAs (acetate, propionate, butyrate) support Treg differentiation via their interaction with the G-protein-coupled receptor (GPR41, GPR43, GPR109A) as well as HDAC inhibition (40).SCFAs ameliorate allergic airway inflammation through sequential induction of myeloid-derived suppressor cells (PMN-MDSCs) and Treg cells (41). A systematic review analyzing 37 studies found that the three major SCFAs in early life had protective effects against allergic diseases, as Butyrate and propionate induced naïve T cell differentiation toward Tregs (42).

Although the environmental factors reviewed in this section are mechanistically diverse, they converge on three shared terminal pathways that collectively drive upper airway dysbiosis and AR susceptibility (Figure 1). The first is the epithelial barrier disruption pathway: pollutants such as PM₂.₅ and DEP generate ROS that degrade tight junction proteins (ZO-1, occludin, claudin-1), physically breaching the nasal mucosal barrier and creating permissive conditions for pathobiont colonization. The second is the immune polarization pathway: DEP, allergens, and climate-driven increases in pollen load activate dendritic cells toward Th2 polarization while suppressing regulatory T cell induction, establishing a pro-allergic immunological milieu that perpetuates dysbiosis. The third is the microbial diversity erosion pathway: early-life antibiotic exposure directly eliminates protective commensals (notably Clostridium clusters IV/XIVa and nasal Corynebacterium spp.), while urbanization-associated loss of environmental biodiversity deprives the developing immune system of the diverse microbial stimulation required for tolerogenic maturation. Understanding these converging pathways provides a unified conceptual model within which disparate environmental risk factors can be systematically integrated, and against which microbiome-targeted preventive interventions can be rationally designed.

## 3 Nasal microbiome in health and allergic rhinitis

### 3.1 Core nasal microbiome in healthy individuals

#### 3.1.1 Dominant Phyla and distribution characteristics

The human nasal microbiome represents a relatively low-diversity ecosystem with distinctive compositional features. 16S rRNA gene sequencing on nasal and oral cavity samples from 12 healthy adults revealed that nasal bacterial communities were dominated by Actinobacteria, Firmicutes, and Proteobacteria (43). These communities were statistically distinct from those on the tongue and buccal mucosa. The same Staphylococcaceae operational taxonomic unit (OTU) was present in all nasal cavity samples, comprising 2.2%–55.0% of the community. Staphylococcaceae was comparatively uncommon in the oral cavity (43).

Analysis of the 347 nasal samples from asthmatics (*n* = 12), allergic rhinitis (AR, *n* = 53), AR with asthma comorbidity (*n* = 183), and healthy control subjects (*n* = 99) revealed that there were four major phyla present in abundance ≥2%: Firmicutes (44.9%), Actinobacteriota (27.7%), Proteobacteria (20.3%) and Bacteroidota (4.6%) (44). The three phyla included 10 dominant genera (abundance ≥2%): Corynebacterium (21.9%), Staphylococcus (18.3%), Dolosigranulum (10.6%), Moraxella (8.8%), Streptococcus (5.2%), Lawsonella (3.9%), Anaerococcus (2.8%), Haemophilus (2.8%), Neisseriaceae sp. (2.7%), and Peptoniphilus (2.4%) (44). Two ASVs – Streptococcus oralis and Staphylococcus aureus comprised the nasal core microbiome (prevalence >90%), accounting for 3.5% and 17.1% of the total reads, respectively (44).

#### 3.1.2 Dolosigranulum pigrum as a candidate nasal probiotic

Among nasal commensal bacteria, Dolosigranulum pigrum has received disproportionate investigative attention for several scientifically justified reasons. First, unlike many other nasal commensals, D. pigrum has demonstrated measurable competitive exclusion of major respiratory pathogens (S. aureus and S. pneumoniae) in controlled *in vitro* experiments. Second, its abundance trajectory—progressively increasing from birth to peak at approximately 12 months of age before declining in adolescence—temporally overlaps with the critical immunological window for allergic sensitization, making it a plausible candidate for early-life immune programming. Third, genomic analysis of multiple strains has begun to elucidate the molecular mechanisms underlying its probiotic potential, providing a more advanced mechanistic evidence base than currently exists for most other nasal commensals. The following section summarizes this evidence while acknowledging that other protective commensals, particularly Corynebacterium pseudodiphtheriticum and Corynebacterium accolens, also play important colonization resistance roles that are discussed in Section 3.2.4.

Dolosigranulum pigrum has gained much interest as a candidate nasal probiotic. D. pigrum is a gram positive, lactic acid bacterium of the phylum Firmicutes, class Bacilli, order Lactobacillales and family Carnobacteriaceae (45), non-sporeforming, facultatively anaerobic, catalase negative, and usually sensitive to the *β*-lactams, clindamycin, and most common antibiotics. Dolosigranulum has a progressive increase in mean relative abundance within the nose from ∼1% post-partum, up to 10%–20% at 12 months of age with little change throughout childhood until declining during adolescence (45).Necessary characteristics of nasal probiotics include the ability to adhere to epithelium and successfully colonize the human upper respiratory tract (URT). They should lack cytotoxicity to respiratory epithelial cells and have low propensity to invade host tissues. Susceptibility to commonly available antibiotics is also required. D. pigrum is increasingly viewed as a keystone species within the human URT and a promising nasal probiotic candidate (45).

*In vitro* experiments and genomic analysis revealed that D. pigrum inhibited S. aureus growth (46). However, effective inhibition of Streptococcus pneumoniae required D. pigrum cooperation with specific nasal Corynebacterium species (such as Corynebacterium pseudodiphtheriticum). The L-lactic acid concentrations produced by D. pigrum were insufficient to explain these inhibitory effects. Genomic analysis of 11 D. pigrum strains revealed relatively small genome sizes (<2 Mb for most strains). Predicted auxotrophies for several amino acids, polyamines, and enzymatic cofactors suggest dependence on the host or other microbes for these nutrients.

It should be noted that protective nasal commensalism is not restricted to D. pigrum. Corynebacterium pseudodiphtheriticum cooperates synergistically with D. pigrum to inhibit S. pneumoniae, and Corynebacterium accolens produces fatty acids that suppress S. aureus growth. The relative abundance of the Corynebacterium genus is consistently lower in AR patients compared with healthy controls, indicating that the broader Corynebacterium–Dolosigranulum commensal community, rather than any single taxon, constitutes the core colonization resistance network of the healthy nasal microbiome.

### 3.2 Dysbiosis patterns in allergic rhinitis patients

#### 3.2.1 Controversy regarding *α*-diversity changes

The changes to *α*-diversity of the nasal microbiome in AR patients are still controversial among different studies. Analysis of 364,923 high quality bacterial 16S rRNA gene sequence reads from 104 middle turbinates mucosa samples (healthy participants *n* = 30, AR patients *n* = 42) noted a significant reduction of species richness in the AR patients (*P* < 0.001) (12). No significant difference of Shannon diversity index were found (*P* = 0.39) (12).

In a meta-analysis of nine studies with a total of 447 AR patients, most studies did not observe any significant change in microbial diversity between AR patients and healthy control groups (47), whereas some studies found greater or lesser species diversity for the AR group. The only common finding among all studies was that AR patients had significantly distinct microbial abundance when compared to healthy control subjects (47).

In another study it is revealed that all alpha diversity indices have been changed significantly (*P* < 0.01) between the AR groups vs. healthy controls, while all of the *β*-diversity indices were different with *P* < 0.011 among each of the respiratory disease groups vs. controls (44).

The inconsistencies in reported findings across AR nasal microbiome studies are substantially attributable to methodological heterogeneity operating at multiple levels. Regarding sampling sites, the anterior nares, middle turbinate mucosa, nasopharynx, and nasal lavage fluid harbor compositionally distinct bacterial communities: the anterior nares are enriched in Staphylococcaceae, the middle turbinate mucosa contains proportionally higher Corynebacterium and Dolosigranulum, and the nasopharynx captures microbial communities more representative of the upper respiratory tract as a whole. Direct comparisons across studies using different sampling sites are therefore methodologically constrained. Regarding sequencing platforms and bioinformatics pipelines, the choice of 16S rRNA hypervariable region (V1–V2 vs. V3–V4 vs. V4) markedly affects genus-level resolution, and differing OTU clustering thresholds and chimera filtering strategies across QIIME, DADA2, and Mothur pipelines can generate substantially divergent community composition estimates from identical samples. Regarding age groups, the nasal microbiome undergoes pronounced developmental transitions: Dolosigranulum pigrum peaks in relative abundance during infancy and declines after adolescence, Moraxella catarrhalis is disproportionately prevalent in young children, and Staphylococcus aureus carriage increases with age. Studies enrolling infants, school-age children, and adults are therefore not directly comparable. Additional confounders include allergen sensitization profile (house dust mite vs. pollen vs. mold), disease severity classification (intermittent vs. persistent; mild vs. moderate-to-severe), geographic region, season of sampling, and concurrent medication use. Establishing standardized sampling protocols, minimum sequencing depth requirements (≥10,000 reads per sample), and shared bioinformatics workflows is essential for generating reproducible, cross-comparable nasal microbiome data in future AR research.

#### 3.2.2 Staphylococcus aureus overgrowth

S. aureus overgrowth in the nasal cavity of AR patients represents an important dysbiosis feature. Pyrosequencing data from 32 subjects demonstrated that healthy participants had greater abundance of S. epidermidis, Corynebacterium accolens, and Nocardia coeliaca, accounting for 41.55% of mapped sequences in nasal mucus (12). In contrast, S. aureus exhibited the greatest abundance (37.69%) in AR patients' nasal mucus. This was associated with positive response to house dust mites (12).

A study of 65 patients with perennial allergic rhinitis (PAR) and 45 non-allergic controls showed that the nasal carriage rate of S. aureus in PAR patients (44%) was significantly higher than in controls (20%, *P* < 0.01) (48). The rate of nasal carriage of superantigen-producing S. aureus in patients (22%) was significantly higher than in controls (6.7%, *P* < 0.05). Nasal symptom scores of S. aureus-positive patients were significantly higher than those of S. aureus-negative patients (*P* < 0.05). Peripheral blood lymphocytes from patients showed significantly higher proliferative responses to superantigens. They were more likely to produce Th2-type cytokines in response to staphylococcal enterotoxin B or toxic shock syndrome toxin 1 (*P* < 0.01). PAR leads to a higher carriage rate of S. aureus, and nasal carriage of S. aureus may aggravate PAR (48).

#### 3.2.3 Moraxella catarrhalis and respiratory disease risk

Moraxella-dominant microbiome signatures correlate to higher risk of respiratory diseases. Six nasal airway microbiome clusters have been observed in 16S rRNA sequenced nose secretion samples collected from 413 asthmatic children (age range: 6–17) and these clusters were dominated by Moraxella, Staphylococcus, Corynebacterium, Streptococcus, Alloiococcus or Haemophilus. Microbiome dominated by Moraxella was linked to higher risk for asthma exacerbation and eosinophil activation. Microbiomes dominated by Staphylococcus or Corynebacterium correlated with decreased respiratory illness/exacerbation events (49). *In vitro* experiments further elucidated the pathogenic mechanisms of M. catarrhalis (49). Compared with other dominant nasal bacterial isolates (S. epidermidis, Corynebacterium propinquum, and Lactobacillus sakei), M. catarrhalis consistently induced significantly greater epithelial cell damage (increased lactate dehydrogenase release). It also induced greater inflammatory cytokine expression (enhanced IL-8 and IL-33 expression) (49).

In a birth cohort, prospective analysis of early nasal microbiota revealed five microbiome profiles that were dominated by Moraxella, Streptococcus, Dolosigranulum, Staphylococcus and Corynebacteriaceae (50). The children in the early Moraxella-dominant profile had the highest ARI rates. Those with Corynebacteriaceae dominant profile had the least (738 vs. 552/100 child-years; unadjusted IRR = 1.34, 95%CI: 1.16–1.54, *P* < 0.001). The association of Moraxella-dominant profile with ARI was still statistically significant after controlling for these nine possible confounding factors (adjusted IRR=1.19, 95%CI: 1.04–1.37, *P* = 0.01) (50).

#### 3.2.4 Loss of protective commensals

The loss of protective commensal bacteria is another microbiome feature associated with AR and related respiratory diseases. One cross-sectional study enrolled children aged 2–18 years admitted for asthmatic exacerbation to the medium care unit (MCU; *n* = 84) or intensive care unit (ICU; *n* = 78), with cases aged 2–6 years (*n* = 87) each matched to two controls (*n* = 182). Cases showed a greater Shannon diversity index (*P* = 0.002) and distinct microbial community composition relative to controls (PERMANOVA R^2^ = 1.9%; *P* < 0.001) (51). Staphylococcus and “oral” taxa including Neisseria, Veillonella, and Streptococcus were enriched in cases, whereas *Dolosigranulum pigrum*, *Corynebacterium*, and *Moraxella* were depleted (MaAsLin2; q < 0.25). Neisseria abundance was associated with greater disease severity (ICU vs. MCU, *P* = 0.03) (51). *D. pigrum* cooperates with *Corynebacterium* species to inhibit respiratory pathogens including *Staphylococcus aureus* and *Streptococcus pneumoniae*, indicating a keystone role in upper respiratory tract homeostasis (46). *D. pigrum* has been further proposed as a nasal probiotic candidate, supported by its consistent inverse association with pathobiont colonization and its capacity to modulate innate immune responses (45).

### 3.3 The dysbiosis-epithelial barrier dysfunction axis

#### 3.3.1 Alarmin release and tight junction disruption

Disruption of nasal epithelial barrier integrity is a critical component of AR pathogenesis. The nasal epithelial barrier comprises cell junctions including tight junctions (TJs), adherens junctions, desmosomes, and hemidesmosomes (17). These constitute the first line of defense against invasion of harmful pathogens or aeroallergens. TJs are composed of transmembrane proteins occludin, claudin, and JAMs, as well as intracellular adaptor proteins ZO. Impairment of TJ molecules plays causative roles in AR pathogenesis (17). AR onset is also triggered by disrupted sinonasal epithelium through production of inflammatory epithelium-derived cytokines TSLP, IL-25, and IL-33 (17). These cytokines are key regulatory factors connecting epithelial-mesenchymal communications. TSLP is an IL-7-like cytokine that potently induces deregulation of Th2 responses. IL-25 (also known as IL-17E) interaction with IL-17RA/B leads to activation of transcription factors. IL-33 is considered a key cytokine that primarily activates type 2 innate lymphoid cells (ILC2s). Th2 cytokines overproduced by various cells suppress transcription of TJ molecules. This causes the breakdown of nasal epithelial barrier from “tight” to “leaky” properties in AR patients.

#### 3.3.2 Nasal microbiome modulation of epithelial immunity

In a study, S. aureus (AR-SA) and S. epidermidis (AR-SE) were isolated from the nasal mucosa of AR patients and their effects on allergic nasal mucosa were examined by using *in-vitro* and *in-vivo* AR model (52). The staphylococcus colonization was more dominant in allergic nasal mucosa. In AR nasal epithelial (ARNE) cell, IL-33 mRNA level was down-regulated after the inoculation of AR-SA and reached its lowest level at 48 h post infection. IL-33 mRNA level also slightly downregulated in AR-SE inoculated ARNE cells at 48 h but inhibition by AR-SA were more prominent. There is no significant change for TSLP mRNA level (52).

Nasal commensal S. aureus from AR subjects mediates anti-allergic effects by modulating IL-33-dependent Th2 inflammation (52). This demonstrates the role of host-bacterial commensalism in shaping human allergic inflammation. Comparing M. catarrhalis with other dominant nasal bacterial isolates, M. catarrhalis consistently induced higher epithelial damage and inflammatory responses (IL-8 and IL-33 expression). S. epidermidis and C. propinquum induced less epithelial damage and cytokine expression (49).

#### 3.3.3 Bidirectional relationship between dysbiosis and barrier dysfunction

A bidirectional relationship exists between dysbiosis and epithelial barrier dysfunction. Dysbiosis leads to barrier dysfunction through multiple mechanisms. Pathogenic bacteria (such as M. catarrhalis) can directly damage epithelial cells and induce expression of inflammatory cytokines including IL-33 and IL-8 (49). Loss of protective commensals (such as D. pigrum and Corynebacterium) weakens colonization resistance against pathogens (46, 51).

Barrier dysfunction also contributes to the establishment of dysbiosis. Tight junction disruption enhances epithelial permeability, which allows for allergen and pathogen penetration through the epithelial barrier (17). Alarmin (TSLP, IL-25, IL-33) release activates Th2 inflammatory response which in turn further alters the local micro-environment. The Th2 cytokines suppress the TJ molecules transcription. This creates a vicious circle (17).

**Figure 2:**
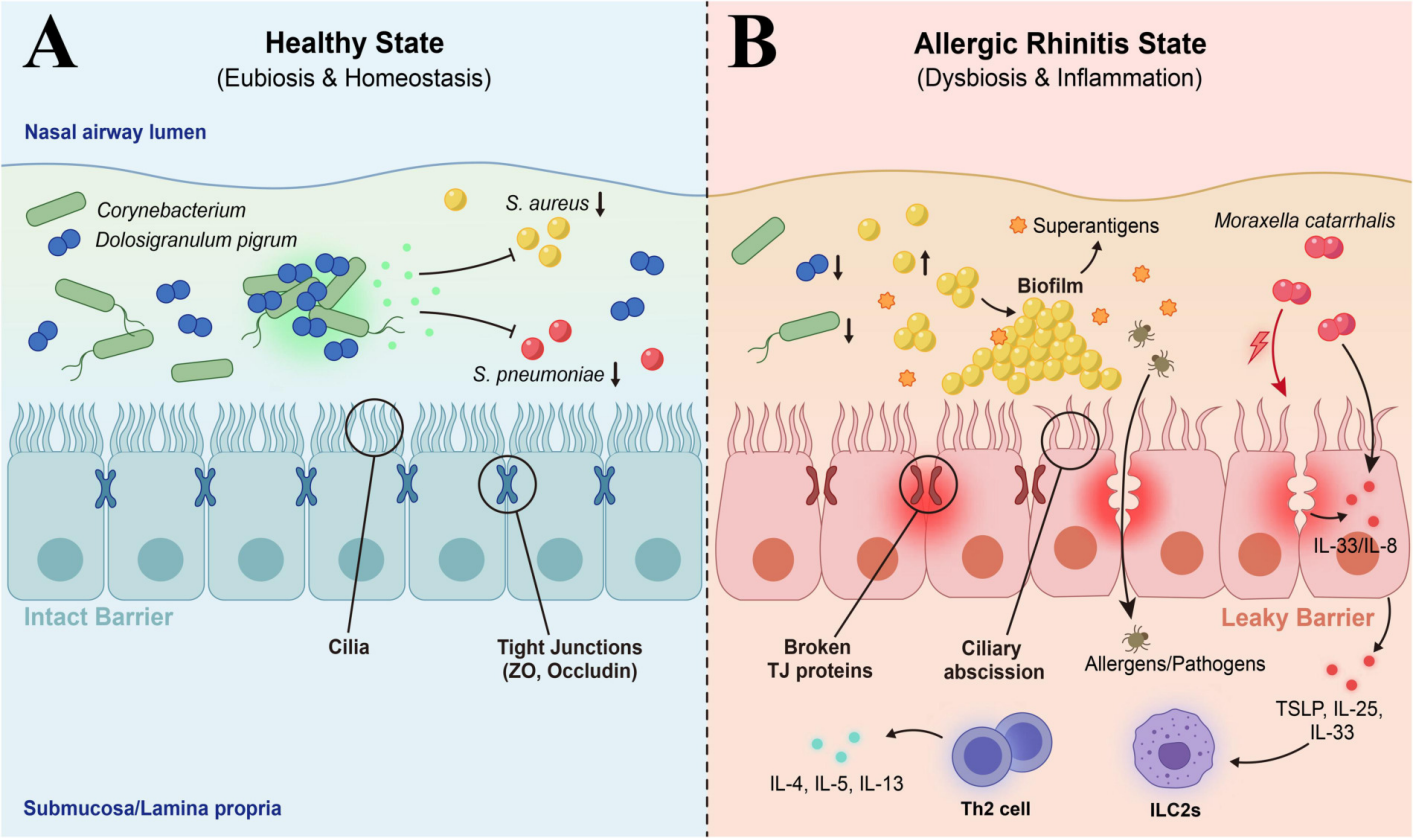
Nasal microbiome dysbiosis and epithelial barrier dysfunction in allergic rhinitis. **(A)** Normal nasal mucosa. Protective commensals (Corynebacterium, Dolosigranulum pigrum), which provide a colonization resistance to both S. aureus and S. pneumoniae. Intact epithelial barrier (tight junctions). **(B)** Allergic rhinitis. Dysbiosis is characterized by loss of protective commensals as well as proliferation of S. aureus and M. catarrhalis. Barrier breakdown results in release of alarmins (TSLP, IL-25, IL-33), that stimulate ILC2s and promote Th2 responses. Th2 cytokines also downregulate TJs to perpetuate the inflammatory response.

In summary, AR patients dysbiosis involves loss of protective commensals (Corynebacterium, D. pigrum) and overgrowth of pathobionts (S. aureus, M. catarrhalis). This dysbiosis leads to disruption of epithelial tight junctions as well as the release of alarmins which initiates an inflammatory response dominated by T helper type-2 (Th2) responses (Figure 2).

## 4 The oral microbiome: an underestimated participant

### 4.1 Introduction: anatomical continuity of the oral-respiratory tract

The mouth is the portal of entry into the respiratory tract and there are intricate relationships between the oral microbiome and lower respiratory tract disease. The human oral cavity hosts the second largest and most diverse microbiota following the gut. Over 700 bacteria have been identified in the oral cavity with representatives on both the hard surfaces (teeth) and the soft tissues of the oral mucosa, developing into a very heterogeneous ecosystem (53, 54).

The anatomical continuity from the mouth through the nose into the nasopharynx and lower airway offer natural routes for microbial traffic. Healthy lung microbiome is associated with a low biomass characterisation. In this case it seems that the microbiome may be more determined by an equilibrium of immigration vs. clearance rather than colonising communities (55). Oral bacteria appear to be the primary route to lower airway entry through microaspiration. This brings the study of the oral microbiome into focus for understanding and studying respiratory diseases.

The oral microbiota of patients with allergic rhinitis has been demonstrated to exhibit significant alterations. The nasal bacteriome of patients with allergic rhinitis and asthma differs from healthy controls at both phylum and genus levels (44). The oral bacteriome of these patients also significantly differs from healthy controls at five phyla and 13 genera (56). These findings provide microbiological evidence for the functional connection of the oral-nasal-lung axis.

**Figure 3:**
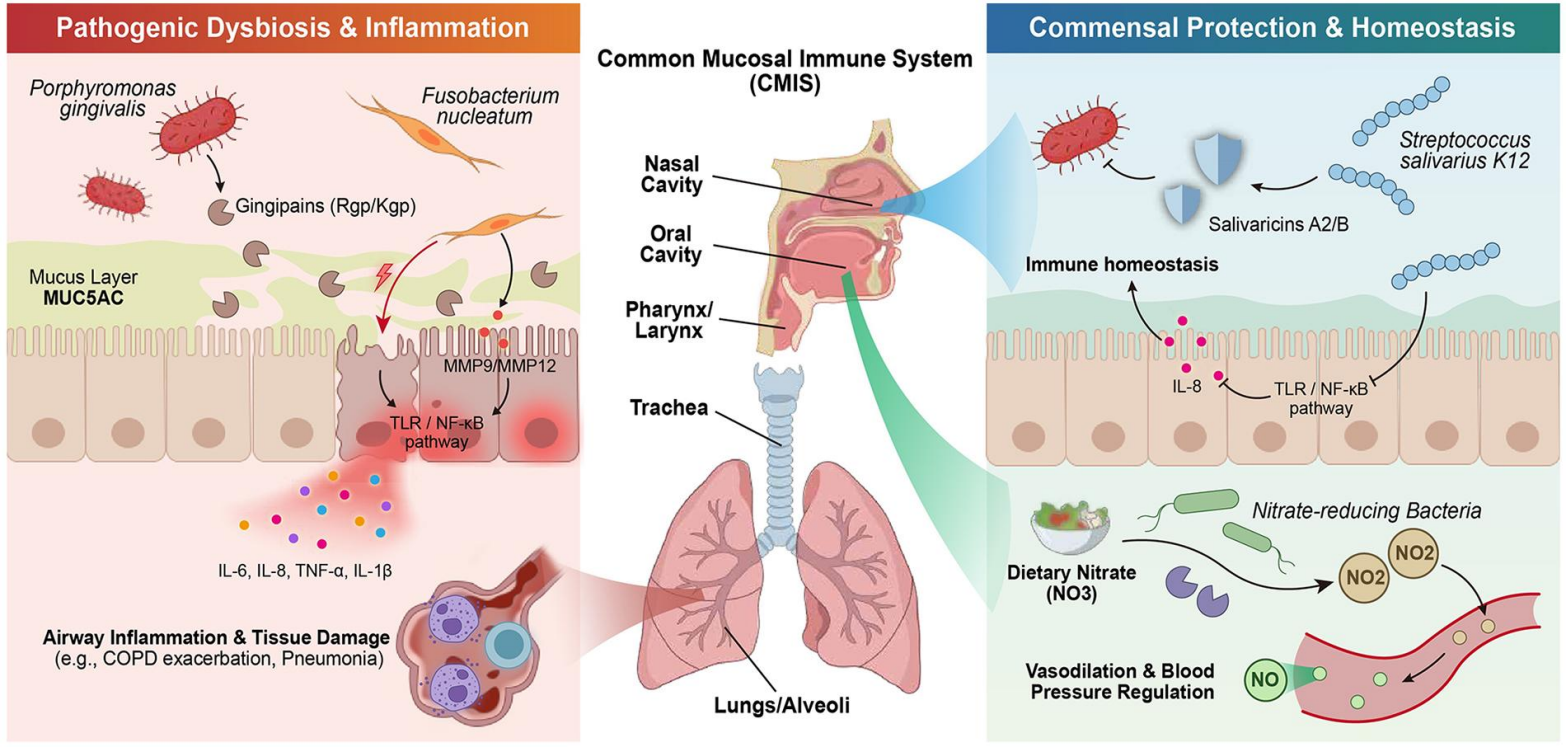
The oral-respiratory axis in respiratory health and disease. Oral microbes migrate to lower airways via microaspiration within the Common Mucosal Immune System (CMIS). (Left) Pathogenic pathways. Periodontal pathogens (P. gingivalis, F. nucleatum) release gingipains to degrade MUC5AC and upregulate MMPs (MMP9, MMP12), and induce the release of pro-inflammatory cytokines (IL-6, IL-8, TNF-α, IL-1β) through the activation of NF-*κ*B. (Right) Protective pathways. S. salivarius K12 inhibits NF-*κ*B translocation, reduces IL-8 secretion, and produces bacteriocins (Salivaricins A2/B). Oral nitrate-reducing bacteria generate nitric oxide for vasodilation. CMIS, Common Mucosal Immune System; MMP, Matrix Metalloproteinase.

The International Consensus Statement on Allergy and Rhinology: Allergic Rhinitis (ICAR-AR 2023), highlights that we should view the upper respiratory tract as one single airway (57), which gives us an idea to understand the role of the oral microbiome in allergic rhinitis pathogenesis. We summarized the functions of the Oral-Respiratory Axis in Figure 3.

### 4.2 Oral pathogens and respiratory inflammation

#### 4.2.1 Porphyromonas gingivalis

Porphyromonas gingivalis is one of the major pathogens of periodontitis. Its unique virulence factors—gingipains—play critical roles in respiratory diseases. Gingipains include arginine-specific proteinases (RgpA and RgpB) and lysine-specific proteinase (Kgp). They represent the most important virulence factors of P. gingivalis (58).

Animal studies have demonstrated that gingipains are not required for P. gingivalis colonization and survival in the lungs. However, they are essential for the manifestation of clinical symptoms and infection-related mortality (59). Pathologies caused by wild-type P. gingivalis W83 included hemorrhage, necrosis, and neutrophil infiltration. These pathologies were absent from lungs infected with gingipain-null isogenic strains. Arginine-specific gingipains made a greater contribution to morbidity and mortality than lysine-specific gingipains (59).

The effect of Gingipains on MUC5AC Gene Expression & Protein Level in Respiratory Epithelial Cells. In mice lungs after treatment with double mutant Kgp/Rgp, there was no increase in MUC5AC level (60). It means that the main factor that influences MUC5AC is the Virulence Factor Gingipains. By this result we can understand the mechanism of action of Periodontal Pathogens to influence Airway Mucus Secretion via Virulence Factors.

P. gingivalis alters lung microbiota composition and aggravates disease severity in COPD rats through upregulation of the Hsp90*α*/MLKL pathway (61). Additionally, P. gingivalis induces airway epithelial cells to produce pro-inflammatory factors including IL-6, IL-8, TNF-α, MCP-1, and CRP (58). Uniquely, P. gingivalis-derived LPS signals through TLR2 (rather than TLR4, as with conventional Gram-negative LPS), inducing a qualitatively distinct NF-*κ*B activation profile with preferential upregulation of IL-6 and IL-8 in airway epithelial cells—a mechanism separable from the ROS-driven NF-*κ*B activation pathway described for PM₂.₅ in Section 2.1.3 (62). In the context of AR, the systemic pro-inflammatory effects of P. gingivalis—mediated through circulating gingipains and lipopolysaccharide—may amplify mucosal Th2 responses in the nasal cavity, potentially worsening AR symptom severity. Epidemiological data suggest higher periodontitis prevalence among AR patients compared with non-atopic controls, though direct mechanistic evidence establishing causality between P. gingivalis nasal colonization and AR exacerbation remains to be demonstrated in prospective human studies.

#### 4.2.2 Fusobacterium nucleatum

Although much of the evidence for F. nucleatum in respiratory disease derives from COPD and pneumonia models, the pro-inflammatory molecular mechanisms it engages—including NF-*κ*B-mediated cytokine induction and epithelial barrier disruption—are broadly relevant to mucosal inflammatory conditions and provide a mechanistic rationale for investigating its potential role in AR. Fusobacterium nucleatum is a “bridging species” in oral biofilms. It plays a central role in interspecies coaggregation. This bacterium has been found to be significantly increased in bronchoalveolar lavage fluid (BALF) and sputum of COPD patients (63).

The effects of F. nucleatum on alveolar epithelial cells have been confirmed by multiple studies. In A549 human alveolar epithelial cells, F. nucleatum (MOI 100) significantly enhanced the secretion of IL-1β, IL-6, and TNF-α (64). Animal experiments demonstrated that after intratracheal administration of F. nucleatum, MMP9 mRNA expression in mouse lung tissue increased by 5.54 ± 1.15-fold. MMP9 protein levels and activity in BALF were significantly elevated (65).

Animal studies further demonstrate that F. nucleatum upregulates MMP9 and MMP12 expression in lung tissue, promoting extracellular matrix degradation—a mechanism with potential relevance to airway mucosal remodeling in upper respiratory inflammatory conditions including AR, although direct evidence in the nasal context is currently lacking. Whether F. nucleatum reaches the nasal mucosa through microaspiration in AR patients, and whether its pro-inflammatory mechanisms documented in COPD and pneumonia models are recapitulated in the nasal epithelial context of AR, represents an important unresolved question warranting dedicated investigation.

#### 4.2.3 Other periodontal pathogens

Other periodontal bacteria implicated in respiratory inflammation alongside *P. gingivalis* and *F. nucleatum* include the “red complex” members *T. forsythia* and *T. denticola* (66, 67). Periodontal bacteria in saliva may access the lower respiratory tract via microaspiration. Under conditions of immunocompromise or impaired mucosal barriers, these organisms may induce or aggravate respiratory infection and inflammation. Mixed infection with *P. gingivalis* and either *T. forsythia* or *T. denticola* synergistically elevates IL-6 production in macrophages, with gingipains serving as a key mechanistic driver (68). All three red-complex species trigger inflammatory cell death and release of endogenous danger signals—including HMGB1 and HSP60—which may amplify pulmonary inflammatory cascades following microaspiration (69).

### 4.3 Protective effects of oral commensal Bacteria

In stark contrast to pathogenic bacteria, certain commensal bacteria in the oral cavity possess important protective functions. Streptococcus salivarius K12 is one of the most extensively studied oral probiotics.

S. salivarius K12 specifically modulates the expression of 565 host genes (70). These genes are involved in multiple innate defense pathways, general epithelial cell function and homeostasis, cytoskeletal remodeling, cell development and migration, and signaling pathways. This strain inhibits baseline IL-8 secretion. It suppresses IL-8 responses to LL-37, Pseudomonas aeruginosa, and flagellin in epithelial cells. Mechanistic studies revealed that K12 exerts immunomodulatory effects by inhibiting NF-*κ*B P65 subunit translocation into the nucleus (70).

S. salivarius K12 produces salivaricin A2 and salivaricin B. These bacteriocins can antagonize multiple respiratory pathogens, providing a molecular basis for its probiotic function (71).

Oral commensal bacteria may protect against CVD by modulating the nitrate-nitrite-NO pathway (57). In a study on 19 healthy volunteers, it was found that following 7 d of using chlorhexidine mouthwash, oral nitrite production decreased by 90% (*p* < 0.001) (72). Plasma nitrite dropped by 25% (*p* = 0.001). Systolic and diastolic BP increased by 2–3.5 mmHg.

The rise in BP was significantly associated with reduced levels of circulating nitrite (r^2^ = 0.56, *p* = 0.002), and the blood pressure effect occurred as early as 1 day after disruption of the oral microflora. The effect persisted throughout the 7 days of the mouthwash treatment (72).

These results demonstrate a clear biological significance for orally resident nitrate-reducing microbiota, which may impact NO levels and BP across the human body.

### 4.4 Immunomodulation of the oral-nasal-lung axis

The oral, nasal, and pulmonary mucosa constitute important components of the Common Mucosal Immune System (CMIS). A review of the 50-year development of the CMIS concept noted three landmark discoveries (73). The discovery of IgA by Tomasi in 1963 was the first. The confirmation of Peyer's patches as an enriched source of precursors for IgA-producing cells by Craig and Cebra in 1971 was the second. The demonstration of bronchus-associated lymphoid tissue by Bienenstock in 1974 was the third (73).

Mucosal immunity is partially compartmentalized. Immunization via nasal mucosa can better stimulate the host's immunity response in upper respiratory tract. Our findings provide some theoretical guidance to design the strategy of oral and nasal probiotics intervention.

Direct evidence linking the oral microbiome to AR pathophysiology is emerging. Pérez-Losada and colleagues demonstrated that the oral bacteriome of patients with AR and asthma differs significantly from healthy controls at five phyla and 13 genera, with enrichment of pro-inflammatory taxa including Prevotella and Veillonella alongside depletion of immunomodulatory species such as Streptococcus salivarius (53). Mechanistically, oral pathogens—particularly Porphyromonas gingivalis—may reach the nasopharyngeal mucosa via microaspiration, whereupon their gingipain virulence factors degrade the MUC5AC mucin layer and activate TLR/NF-*κ*B signaling to induce IL-33 and IL-8 secretion, cytokines centrally implicated in AR mucosal inflammation. Furthermore, oral nitrate-reducing bacteria regulate systemic and local nitric oxide (NO) bioavailability; disruption of this pathway by oral dysbiosis may alter nasal vascular permeability and mucosal inflammatory tone, representing a direct biochemical channel through which oral microbial composition influences nasal immune homeostasis. Collectively, these findings position the oral microbiome not merely as a passenger but as an active contributor to AR pathophysiology through the oral-nasal-pulmonary mucosal continuum.

The oral microbiota modulates the host immune response in various ways. For example, pathogenic bacteria like P. gingivalis stimulate TLR signaling pathways by its virulence factors (gingipains, LPS, fimbriae, etc.) which leads to the activation of NF-*κ*B and pro-inflammatory cytokines; while other commensal bacteria like S. salivarius have an immuno-modulating effect that blocks the NF-*κ*B cascade and modulates genes in the host (59).

## 5 Mechanistic pathways: from dysbiosis to allergic inflammation

### 5.1 Metabolite-mediated immune regulation

**Figure 4:**
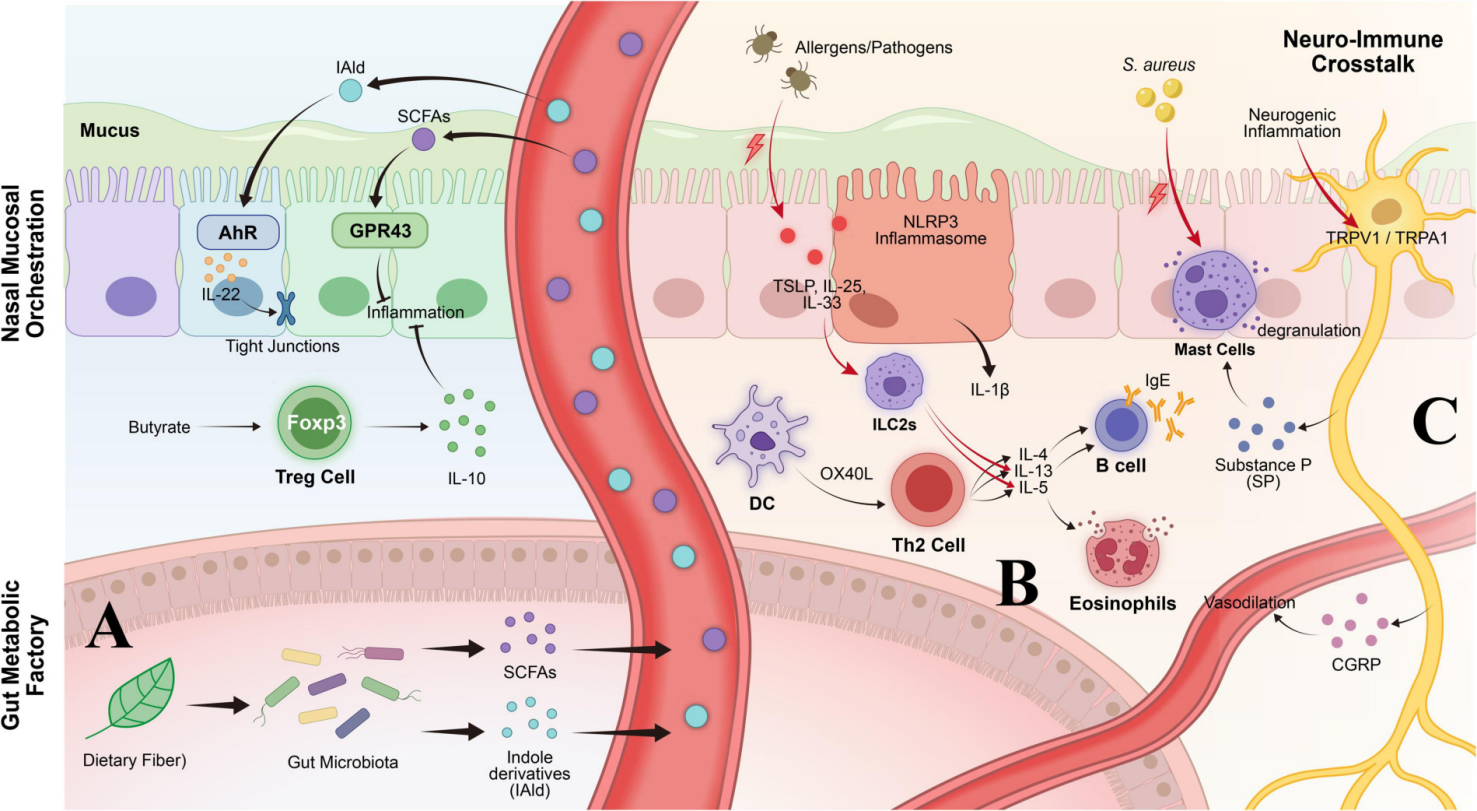
Integrated mechanisms linking microbiome dysbiosis to allergic inflammation. **(A)** Metabolic immunomodulation. Gut microbiota-derived SCFAs inhibit HDACs and promote Treg differentiation. Tryptophan-derived indoles activate AhR, strengthening epithelial barrier and inhibiting NLRP3 inflammasome. **(B)** Immune dysregulation. Dysbiosis triggers alarmin release (TSLP, IL-25, IL-33), activating ILC2s and Th2 cells. Type 2 cytokines drive IgE production and eosinophil infiltration. S. aureus *δ*-toxin induces mast cell degranulation. **(C)** Neuro-immune crosstalk. TRPV1/TRPA1 activation on sensory neurons releases neuropeptides (Substance P, CGRP), promoting vasodilation and mast cell activation via NK-1R/MRGPRX2. The bidirectional arrows between the gut metabolic factory (lower left) and nasal mucosal orchestration (upper left) represent the gut-nasal axis, through which circulating SCFAs and indole derivatives modulate upper airway immune homeostasis. The right-side neuroimmune crosstalk panel represents a third mechanistic dimension through which dysbiosis-driven neurogenic inflammation amplifies Th2 responses and sustains AR symptomatology. Together, these three modules—metabolic, immunological, and neuroimmune—constitute an integrated pathophysiological model of microbiome-to-allergy signal transduction in AR.

Gut microbiota generates various kinds of bioactive metabolites by metabolizing indigestible food for hosts, which plays key roles on immune homeostasis and allergic inflammation regulation (74). The major microbiota derived-metabolites are short chain fatty acids (SCFAs), tryptophan metabolites, and pathogen-specific toxins. They affect immune cell function and barrier integrity in a variety of different molecular ways. The integrated interplay among these pathways is illustrated in Figure 4, which unifies the gut metabolic factory (SCFA and indole derivative production), nasal mucosal immune orchestration (AhR/GPR43 activation, Treg induction, NLRP3 inflammasome regulation), and neuroimmune crosstalk (TRPV1/TRPA1–mast cell axis) within a single conceptual framework. The left panel of Figure 4 depicts how gut microbial metabolites translocate via the circulation to modulate nasal mucosal immunity; the central panel shows how allergen/pathogen sensing at the epithelial surface triggers ILC2 and Th2 activation; and the right panel illustrates how sensory neuron activation amplifies and sustains the allergic inflammatory state through neuropeptide release.

#### 5.1.1 Short-chain fatty acids (SCFAs)

Short-chain fatty acids are the primary products of gut microbial fermentation of dietary fiber and resistant starch. They mainly comprise acetate, propionate, and butyrate (75). High-fiber diets promote the proliferation and metabolic flux of SCFA-producing microbes (74). These metabolites serve as the principal energy source for colonocytes. They also serve as critical signaling molecules for immune regulation.

##### 5.1.1.1 Immunomodulatory mechanisms of butyrate

Butyrate, one of the most biologically active SCFAs, has immunomodulatory activity that largely depends on its ability to inhibit histone deacetylases (HDAC). IL-4 inhibits regulatory T cell (Treg) differentiation via a STAT6 dependent mechanism as well as by histone deacetylation of the Foxp3 locus mediated by HDAC9. Sodium butyrate, acting as pan-HDAC-inhibitor, efficiently reverses the IL-4-mediated inhibition and ameliorates allergic airway inflammation in mice (76).

In allergic airway inflammation models, exogenous SCFA treatment exerts protective effects through sequential induction of polymorphonuclear myeloid-derived suppressor cells (PMN-MDSCs) and Treg cells. Mice receiving SCFAs developed less severe asthma accompanied by expansion of PMN-MDSCs and Treg cells. Depletion of PMN-MDSCs abrogated the protective effects of SCFAs (41).

##### 5.1.1.2 Anti-inflammatory effects of propionate and acetate

The main immunomodulatory effects of propionate are mediated by GPR43 (FFAR2), while those of acetate are mediated mainly by GPR43 and GPR41 (FFAR3) (77). Propionate binds to GPR43. SCFAs modulate inflammasome activity: they promote IL-22 secretion from GPR43 expressing group 3 innate lymphoid cells (ILC3s) thus promoting intestinal barrier integrity (78). SCFAs also promote B cell differentiation, as well as mucosal IgA production, modulating APCs phenotypes, promoting immunotolerance and restraining overreactive inflammation (79).

##### 5.1.1.3 Allergic rhinitis and Gut SCFA-producing bacteria

In gut microbiome research on AR, it has been shown that the abundance of bacteria producing SCFAs was significantly reduced (47). In a systematic review, it was shown that the abundances of SCFA-producing genera such as Blautia, Eubacterium hallii, Romboutsia, Collinsella, Dorea (47)—fecal SCFA level was positively associated with abundance of those genera. In terms of the nasal microbiome, allergic rhinitis patients have been shown to experience a Staphylococcus aureus dominant dysbiosis with an increase in the relative abundance of Staphylococcus (12).

In gut lung axis researches, it has been shown that the SCFAs from the gut enter into lungs via circulatory system. The SCFAs dampen allergic airway inflammation by suppressing Th2 response mediated through GPR41/43 as well as inhibiting HDACs resulting in expansion of Tregs cells (80). Ingestion of high fiber diet enhances blood SCFA level and lowers down the prevalence of allergic airway disease which can be correlated to enhancement of haematopoeisis (81).

#### 5.1.2 Tryptophan metabolites

Tryptophan (Trp) is an essential amino acid. It can be metabolized by host and microbial enzyme systems into various immunomodulatory metabolites (82). Gut microbiota-produced tryptophan metabolites play crucial roles in regulating mucosal immune responses through interaction with the aryl hydrocarbon receptor (AhR).

##### 5.1.2.1 Indole derivatives and aryl hydrocarbon receptor activation

Intestinal lactobacilli can metabolize tryptophan to indole-3-aldehyde (IAld). IAld activates AhR to promote AhR-dependent transcription of IL-22, thereby maintaining intestinal mucosal homeostasis and antifungal defense (83). Three tryptophan metabolites—indole-3-ethanol (IEt), indole-3-pyruvate (IPyA), and IAld—reduce inflammatory cytokine-induced intestinal permeability through AhR-dependent mechanisms. They maintain the integrity of tight junction complexes and actin-regulatory proteins (84).

##### 5.1.2.2 Skin microbial tryptophan metabolism and atopic dermatitis

Skin microbes can also metabolize tryptophan to generate indole derivatives which activate the epidermal AhR. Majority of bacteria isolated from human skin can metabolize tryptophan and activate AhR (85). IAld level is much less abundant in either lesional or non-lesional skin of AD patient when compared with normal subjects. IAld topical application significantly ameliorated MC903 induced AD like inflammation in a AhR dependent manner. This includes AhR binding to the TSLP promoter in order to down-regulate TSLP expression (86).

#### 5.1.3 Pathogen-derived metabolites

Staphylococcus aureus colonizes lesional skin in more than 90% of atopic dermatitis patients (87). S. aureus-produced *δ*-toxin is a potent inducer of mast cell degranulation. *δ*-toxin-induced mast cell degranulation depends on phosphoinositide 3-kinase (PI3 K) and calcium (Ca^2^^+^) influx. Unlike IgE crosslinking-mediated degranulation, it does not require spleen tyrosine kinase (Syk) (87).

IgE potentiates *δ*-toxin-induced mast cell degranulation in the absence of antigen. Skin colonization with S. aureus induces IgE and IL-4 production as well as inflammatory skin lesions. In mast cell deficient Kit^(W-sh/W-sh) mice, the effects of *δ*-toxin in terms of IgE production and dermatitis are completely abolished. These effects are recovered with mast cell reconstitution (87). MRGPRX2 is involved in pruritic symptoms of AD by *δ*-toxin-induced mast cell degranulation (88).

Staphylococcus aureus expresses multiple virulence factors. These include superantigens (SEA, SEB, TSST-1), phenol-soluble modulins (PSM*α* and PSM*β*), *α*-toxin, and *δ*-toxin. Superantigens activate basophils and cause T cell-mediated inflammation. The PSM family stimulates keratinocytes to release pro-inflammatory cytokines. *α*-toxin induces keratinocyte cytotoxicity. *δ*-toxin induces mast cell degranulation (89).

### 5.2 Pattern recognition receptor signaling

The innate immune response detects microbial-associated molecular patterns (MAMPs), using pattern recognition receptors (PRRs). These events initiate signaling pathways to regulate inflammatory responses and adaptive immunity, with dysregulated PRR signaling in dysbiosis playing an important role in the development and perpetuation of allergic inflammation.

Toll-like receptors are the major PRR family that recognizes microbial components. TLR2 recognizes lipoteichoic acid and lipopeptides from Gram-positive bacteria. TLR4 primarily recognizes lipopolysaccharide (LPS) from Gram-negative bacteria (90). In murine allergic rhinitis models, immunization with Streptococcus pneumoniae *Δ*pep27 downregulates TLRs and NLRP3 expression. This alleviates ovalbumin-induced allergic rhinitis symptoms (91).

The NLRP3 inflammasome is a multiprotein complex composed of NLRP3 protein, ASC adaptor protein, and caspase-1. It plays a central regulatory role in allergic inflammation (92). It should be noted that direct clinical evidence for NLRP3 inflammasome activation specifically in AR nasal mucosa remains limited, with most mechanistic data derived from asthma and atopic dermatitis models. However, given the “united airway” concept and the shared epithelial-ILC2-Th2 inflammator*y* axis between AR and asthma, NLRP3-mediated IL-1β and IL-18 maturation in response to allergen and pathogen-associated molecular patterns is mechanistically plausible in nasal mucosa and may represent a conserved mucosal inflammatory mechanism operative across upper and lower airway allergic disease.

NLRP3 inflammasome activation involves a two-step process. First, a priming signal induced by TLR-NF-*κ*B pathways or cytokine signaling upregulates transcription of NLRP3 and pro-IL-1β. Second, an assembly signal triggered by multiple stimuli promotes the assembly of NLRP3, ASC, and caspase-1. These stimuli include K^+^ efflux, Ca^2^^+^ flux, lysosomal destabilization, ROS production, and mitochondrial dysfunction. This ultimately leads to maturation and release of IL-1β and IL-18 and gasdermin D-mediated pyroptosis (93).

NLRP3 inflammasome activity is implicated in type 2 inflammatory disorders including asthma, allergic rhinitis or atopic dermatitis (94), which develop after allergen exposure. NLRP3 requires two steps of activation for the formation of active inflammasomes, which then activates caspase-1 and promote IL-1β and IL-18 maturation. The dysfunction of NLRP3 could cause pyroptosis and enhance local inflammation response (95).

In allergic asthma models, house dust mite inhalation promotes pulmonary NLRP3 inflammasome activation. Treatment with the NLRP3 inhibitor RRx-001 significantly reduces airway inflammatory cell infiltration and mucus secretion. This indicates that NLRP3 promotes allergic asthma development in an inflammasome-dependent manner (96).

### 5.3 Adaptive immune dysregulation

Dysbiosis has profound effects on the adaptive immune system, influencing APC function as well as the cytokine environment; this results in increased Th2 skewing, impaired Treg function, and innate lymphoid cell activation.

#### 5.3.1 Th2 polarization

Type 2 helper T cell (Th2) immune responses represent the core pathological mechanism of allergic diseases. In allergic inflammation, Th2 cells secrete characteristic cytokines including IL-4, IL-5, IL-9, and IL-13. These cytokines drive pathological processes such as IgE class switching, eosinophil activation, and mucus hypersecretion (97).

Epithelium-derived alarmins (TSLP, IL-25, and IL-33) activate dendritic cells. They upregulate expression of pro-Th2 differentiation co-stimulatory molecules such as OX40L. TSLP-activated dendritic cells induce inflammatory Th2 cell responses through OX40 ligand (98).

IL-4 and IL-13 are hallmark Th2 cytokines. They are the primary drivers of B cell IgE class switching. IL-5 is the major growth, differentiation, and survival factor for eosinophils. It promotes eosinophilia and tissue eosinophil infiltration (99).

#### 5.3.2 Treg/Th17 imbalance

Regulatory T cell/T helper 17 cell (Treg/Th17) ratio is essential to maintain the immune system homeostasis, and gut microbiota contributes significantly to maintenance of Treg/Th17 balance and reduction of allergic airway inflammation (80).

Gut microbiota diversity is closely associated with peripheral Treg development. Butyrate-producing bacteria promote Treg development through SCFA-HDAC inhibition mechanisms. This enhances histone acetylation at the Foxp3 locus (100). Clostridia species play a special role in inducing colonic Tregs (39).

ROR*γ*t^+^Foxp3^+^ Tregs are a special Treg population of peripheral origin, which is important for maintaining tolerance towards the gut microbiota. ROR*γ*t^+^Foxp3^+^ Tregs require high dependency from microbial signals for their development and maintenance (101). The polysaccharide A (PSA) produced by Bacteroides fragilis acts directly on T-cells via TLR2 signalling that drive Treg induction and expansion and suppress pro-inflammatory Th17 cell differentiation (102).

Segmented filamentous bacteria (SFB) can induce Th17 cell differentiation in the intestinal lamina propria through direct adhesion to intestinal epithelial cells. SFB antigens are presented by intestinal dendritic cells, driving mucosal Th17 cell differentiation (103).

#### 5.3.3 Group 2 innate lymphoid cell (ILC2) activation

Group 2 innate lymphoid cells (ILC2s) are innate immune cells that lack conventional lineage markers. They express the Th2 cell-associated transcription factor GATA-3. They can produce type 2 cytokines such as IL-5 and IL-13, playing a key amplifying role in allergic inflammation (104). Epithelium-derived alarmins—TSLP, IL-33, and IL-25—are the primary drivers of ILC2 activation. These three alarmins can activate ILC2s individually or synergistically. They induce production of type 2 cytokines including IL-5 and IL-13 (105). In murine asthma models, simultaneous blockade of all three alarmins produces significantly amplified inhibitory effects (106).

TSLP and IL-33 signaling reciprocally enhance each other's lung protein release and expression. They mutually increase the expression of each other's receptors on ILC2s (107).

Early postnatal skin microbial colonization primes skin ILC2s through induction of TSLP expression. The indole-3-aldehyde-producing tryptophan metabolic pathway participates in TSLP-mediated ILC2 priming. This early physiological priming process is co-opted by allergic inflammation pathology in adulthood, amplifying type 2 immune responses (108).

Epithelial alarmin-targeting biologics are now in the clinic. Tezepelumab (anti-TSLP antibody) lowers exacerbations in adults with uncontrolled asthma (109) and dupilumab targeting IL-4 receptor *α* subunit was approved for atopic dermatitis treatment in 2017, tralokinumab (anti-IL-13) in 2021, lebrikizumab (anti-IL-13), which was approved in 2023, and nemolizumab (anti-IL-31) expected to be approved in 2024 (110).

### 5.4 Neuroimmune interactions

Bidirectional communication between the nervous and immune systems plays an important regulatory role in the development and progression of allergic inflammation. Sensory neurons participate in the initiation and amplification of inflammatory responses through neuropeptide release and ion channel activation. This forms “neurogenic inflammation” (111).

Transient receptor potential (TRP) channels are a superfamily of non-selective cation channels. TRPV1 and TRPA1 expression on sensory nerve endings and various immune cells makes them key mediators of neuro-immune crosstalk (112).

TRPV1 (the capsaicin receptor) can be activated by high temperatures (>43 °C), capsaicin, and various endogenous inflammatory mediators (113). TRPV1 activation at sensory nerve endings causes rapid intracellular Ca^2^^+^ elevation. This triggers release of neuropeptides such as substance P (SP) and calcitonin gene-related peptide (CGRP). Neurogenic inflammatory manifestations follow, including vasodilation, plasma extravasation, and leukocyte infiltration (114).

TRPA1 may also be triggered by other chemical stimuli such as acrolein, formaldehyde from tobacco smoke or reactive oxygen species. In Trpa1⁻/⁻ mice, neurogenic plasma extravasation cannot be induced by intratracheal instillation of cigarette smoke extract, indicating that the interaction of electrophilic components of cigarette smoke with TRPA1 is the main mechanism for cigarette smoke-induced acute airway neurogenic inflammation (115).

While the evidence for TRPA1 involvement in AR is currently indirect—primarily inferred from the nasal hyperreactivity phenotype characteristic of AR patients and from shared airway sensory neurobiology—the heightened sensitivity of nasal sensory endings to chemical irritants (formaldehyde, acrolein, SO₂) that are canonical TRPA1 agonists is consistent with TRPA1-mediated neurogenic inflammation contributing to AR symptom amplification. Dedicated studies examining TRPA1 expression and function specifically in AR nasal sensory neurons are needed to establish direct mechanistic evidence.

GDC-0334 is an extremely potent, selective, oral bioavailable antagonist of the TRPA1 receptor. In several different preclinical models, GDC-0334 blocks TRPA1 activity in airway smooth muscle and sensory nerves. It alleviates edema, dermal blood flow, cough, and allergic airway inflammation. GDC-0334 reduced TRPA1 agonist-induced dermal blood flow in a healthy volunteer Phase 1 study, pain, and itch. This demonstrated target engagement in humans (116).

Substance P is one of the members of tachykinin neuropeptide family. It acts mainly by interacting with neurokinin-1 receptor (NK-1R) and MRGPRX2 receptor on its target cell. On mast cells, substance P activates the NK-1R and MRGPRX2 receptor to cause degranulation and aggravate inflammation (117).

CGRP is one of the most abundant neuropeptides. It is widely expressed in the peripheral and central nervous systems. CGRP is one of the most potent vasodilators. It induces vasodilation through action on CGRP receptors on vascular smooth muscle cells (118).

In allergic rhinitis patients, repeated allergen exposure leads to enhanced sensitivity of sensory nerve endings to stimuli (peripheral sensitization). Central nervous system processing of afferent signals also changes (central sensitization). This dual sensitization results in lowered sneeze reflex thresholds. Patients develop sneeze responses to normally innocuous stimuli (119).

## 6 Clinical translation and therapeutic prospects

### 6.1 Microbiome-Based biomarkers

The characteristic changes of nasal microbiome are potential new biomarkers to diagnose AR. The dysbiotic nasal microbiome is dominated by Staphylococcus aureus in patients with AR (12). Using an *in vitro* culture system and animal model, we demonstrated that S. aureus isolated from patients with AR was able to alter Th2 cytokine expression levels in nasal epithelial cells; this indicates that it has regulatory roles during AR pathogenesis.Conversely, certain commensal bacteria may exert protective effects. Dolosigranulum pigrum can inhibit S. aureus growth *in vitro* through competitive mechanisms (46).

A study combining 16S rRNA sequencing and serum metabolomics analyzed samples from 28 AR patients and 15 healthy controls (120). The AR group showed elevated Actinobacteria phylum abundance and significantly increased abundance of seven genera (Klebsiella, Prevotella, Staphylococcus, etc.). Pelomonas abundance was decreased. A random forest prediction model based on ten genera features demonstrated the diagnostic potential of airway microbiome for AR.

The microbiome profile may be used to predict the response to AR treatment. Multiple cytokine profiling in 72 children with house dust mite induced AR who received subcutaneous immunotherapy (SCIT), identified a combination of biomarkers which predicted for effective response to SCIT (121). The serum metabolomics analyses of AR patients treated by SLIT, identified metabolite markers predictive for the efficacy of SLIT (122).

The URECA cohort study evaluated nasal microbiome development dynamics in high-risk urban children during the first three years of life (123). Moraxella and Haemophilus enrichment was associated with high-wheeze phenotypes. These findings suggest that early nasal microbiome characteristics may predict subsequent respiratory disease development.

Microbiome-based endotype classification can optimize the precision diagnosis and treatment of AR. ML methods were applied for endotype AR in children, setting the stage for individualized therapies (124). Stratification approaches based on the microbiome could reveal subsets of patients who are likely to benefit from particular treatments.

Standardizing clinical application of microbiome biomarkers is difficult as there are differences among the various studies with respect to sampling methods, sampling sites, sequencing platforms, and bioinformatics analysis pipeline. Establishing standard sampling criteria, standards of sequencing protocols as well as data analysis workflows is crucial to advance microbiome biomarker clinical translation.

### 6.2 Microbiome-targeted intervention strategies

Nasal direct microecological modulation may be an option for AR therapy. In a randomized, placebo-controlled, crossover trial, a nasal probiotic consortium was tested on 24 patients with seasonal AR (125). The consortium included Lactobacillus rhamnosus SP1, L. paracasei 101/37, and Lactococcus lactis L1A. With the use of nasal allergen challenge model, primary efficacy endpoints (Mini-RQLQ, TNSS, PNIF, FeNO) did not differ significantly from placebo. But this treatment was shown to have a good safety. The present study shows some key evidence on the feasibility and safety of nasal probiotics.

Prebiotics indirectly modulate gut microecology by selectively promoting beneficial bacterial growth. The International Scientific Association for Probiotics and Prebiotics (ISAPP) consensus statement defined the concept and scope of prebiotics (126). Galacto-oligosaccharides (GOS), fructo-oligosaccharides (FOS), and inulin can be specifically fermented by Bifidobacterium and Lactobacillus. This produces short-chain fatty acids (SCFAs).

Postbiotics are defined as microbially derived metabolites, or lysates that have an immunomodulatory effect in the absence of viable bacteria. SCFAs bridge diet, microbiome, and immunity via particular molecular mechanisms (127). Butyrate, propionate, and acetate modulate the differentiation and functioning of regulatory T cells by acting through the activation of a signaling pathway involving GPR41/GPR43 and inhibiting HDACs.

Bacterial lysates (e.g., OM-85) have accumulated substantial evidence in respiratory infection prevention. OM-85 affects both gut and nasopharyngeal microbiomes in preschool children (128). Experimental evidence supports OM-85 in preventing viral respiratory infections (129).

Synthetic biology technology enables construction of engineered bacterial strains with specific functions. IL-10-secreting engineered Lactococcus lactis effectively treats murine colitis models (130). Oral IL-10-secreting L. lactis prevents IgE sensitization in a murine food allergy model (131).

Intranasal administration of recombinant IL-10-expressing L. lactis modulates acute allergic airway inflammation in a murine model (132). A thymidine-auxotrophic L. lactis strain has been developed as a biological containment system. This enhances engineered bacteria safety (133).

The fecal microbiota transplantation (FMT) is the basic strategy to reconstruct the gut microecology. The FMT can alleviate AR by many ways (134), including modulating CD4^+^ T cell subsets, re-establishing gut microbiota diversity, and suppression of the PI3 K/AKT/mTOR and NF-*κ*B signalling pathways. For clinical use in FMT there remains a need to standardize the process of donor selection, preparation stability, and long term safety monitoring.

Despite the scientific promise of these microbiome-targeted strategies, several systematic barriers impede their clinical translation. Evidence gap: The only published nasal probiotic RCT in AR enrolled 24 participants, achieved no significant improvement in primary endpoints, and had a follow-up of only weeks (127); no Phase III nasal microbiome intervention trial exists in AR. For oral probiotics, while a meta-analysis of 28 studies reported modest benefits on RQLQ scores and Th1/Th2 ratios, substantial heterogeneity across strains, dosages, and patient populations limits the generalizability of pooled estimates. Interindividual variability: Host genetic background (HLA type, secretory IgA genotype), prior antibiotic history, diet, and age profoundly influence both baseline microbiome composition and the colonization efficiency of exogenous probiotics, making uniform treatment responses across patients unlikely. Nasal colonization challenges: Unlike the intestinal environment, the nasal cavity presents a high mucociliary clearance rate, active immune surveillance, and limited colonization niche availability for exogenous bacteria, resulting in transient rather than durable microbial engraftment following intranasal probiotic administration. Sustained efficacy may therefore require frequent dosing schedules with inherent compliance challenges. Safety of engineered bacteria: IL-10-secreting Lactococcus lactis and similar constructs, while efficacious in murine models, face regulatory and safety hurdles including horizontal gene transfer risk, behavior in immunocompromised hosts, and long-term ecological consequences that remain inadequately characterized in human trials. Strain-specificity: Probiotic efficacy is highly strain-dependent; extrapolation of findings from one strain to others within the same species is not scientifically justified, and regulatory authorities increasingly require strain-specific clinical data. Overcoming these barriers will require large-scale, multi-center adaptive trial designs with individual microbiome profiling at baseline to identify predictors of treatment response.

### 6.3 Precision medicine

The exposome concept emphasizes systematic evaluation of all environmental factors affecting AR. Climate change alters the exposome and affects allergic disease incidence and severity (135). Relevant factors include temperature, humidity, air pollution, pollen, and gut microbiome.

Combined genomic, transcriptomic, metabolomic and microbiomic information provides a comprehensive view into the pathophysiology of AR. For instance, an integrative analysis of the airway microbiome with serum metabolomics found that there were 26 differential metabolites and 16 perturbed metabolic pathways (120). An analysis of associations between the microbiota and metabolites suggested potential biomarkers for diagnosis.

Artificial intelligence and machine learning (ML) technologies demonstrate significant potential in AR prediction, diagnosis, and personalized management. XGBoost algorithms have been applied to predict AR risk in preschool children (136), and sequential models (LSTM and SLAC) achieve 66%–84% accuracy in predicting subcutaneous immunotherapy adherence (137). However, constructing clinically reliable ML predictive tools requires careful attention to the following key requirements. Regarding feature variables, optimal models should integrate multi-domain inputs including microbiome features (*α*/*β*-diversity indices, relative abundances of key taxa such as S. aureus, D. pigrum, and Moraxella), metabolomic features (fecal and serum SCFA profiles, tryptophan metabolite signatures), immunological features (serum total and specific IgE, peripheral eosinophil counts, cytokine panels including IL-4, IL-5, IL-13, and IFN-*γ*), clinical features (symptom scores, sensitization profiles, disease duration, medication history), and host genomic features (AR-associated single-nucleotide polymorphisms). Regarding dataset requirements, reliable microbiome-based ML models typically require ≥500 samples with standardized collection and sequencing protocols, and external validation in independent cohorts from different geographic regions is essential before clinical deployment. Regarding interpretability, clinical translation demands preference for interpretable algorithms (logistic regression, decision trees with SHAP value analysis) over black-box models, to satisfy transparency requirements for clinical decision-making. The primary current bottleneck is the absence of large-scale, multi-center prospective cohorts with harmonized multi-omics and clinical data—a foundational infrastructure investment required to realize the potential of ML-driven AR precision medicine.

The GUSTO cohort study tracked nasal microbiota establishment during the first 18 months of life (138). Corynebacteriaceae and Staphylococcaceae were associated with health status. Oxalobacteraceae and Aerococcaceae were associated with rhinitis risk.The URECA cohort study evaluated nasal microbiome developmental dynamics in high-risk urban children (123). Further analysis of longitudinal rhinitis phenotypes in the URECA cohort provided additional insights (139). Linkage disequilibrium score regression (LDSC) and Mendelian randomization meta-analysis validated causal relationships between specific gut microbiota and AR risk (140). Evidence supports a causal association between Ruminococcus gauvreauii group and increased AR risk (OR = 1.26) (141).

## 7 Conclusion

This review has attempted to integrate and summarize available information regarding the role of nasal-oral microbiome axis in AR pathogenesis, explaining how interactions between environment, microbiome dysbiosis, and allergic inflammation.

### 7.1 Environmental factors reshape upper respiratory microbiome

A few key environmental variables have a significant impact on the structure and function of the upper respiratory microbiome. Inhalation of airborne pollutants (e.g., PM₂.₅) impacts the integrity of nasal epithelium through disruption in tight junction protein by producing ROS. It has been demonstrated to downregulate ZO-1, occludin, and claudin-1 expression. Pro-inflammatory cytokine release is also increased (15). Exposure to antibiotics in early life is a high-credibility risk factor for AR (Class II). The mechanism appears to be disruption of normal gut microbiome colonisation (14). Biodiversity hypothesis suggests that exposure to nature increases the biodiversity in our body (microbiomes), and it supports immunity. So it prevents allergic condition, while biodiversification decrease by urbanization has a strong correlation with microbiome change and immune system failure (6).

### 7.2 Characteristic patterns of dysbiosis

In AR, the microbiome becomes dysbiotic with loss of protective commensals and an overgrowth of pathobionts. In healthy people, nasal commensals such as Corynebacterium or S. epidermidis predominate in humans. The presence of S. aureus overgrowth has been shown in AR. Its abundance was correlated to HDM sensitisation and patient age (12). D. pigrum is a potential nasal probiotic candidate which may be used for blocking pathogen colonisation via competition. Abundance in the nose is greater in infancy and childhood. Abundance is related to decreased risk of respiratory infections (45).

### 7.3 Pathways linking dysbiosis with allergy and inflammation

The microbiome influences allergic inflammation by a variety of different mechanisms, many of which are interdependent on each other. SCFAs influence the integrity of the epithelium as well as mucosal immune responses by stimulating G-protein coupled receptors or inhibiting histone deacetylases. Butyrate also influences the maturation of intestinal epithelial cells, phagocytes, B-cells, and regulatory T-cells (127). At the adaptive immunity level, dysbiosis favors Th2 polarization and Treg/Th17 imbalance via epithelial alarmin release. The oral microbiota has both local and systemic immunomodulatory activity by means of the oral-nasal-pulmonar*y* axis.

### 7.4 Translational opportunities

The microbiome presents interesting opportunities for both diagnosis and treatment of AR. One recent meta-analysis of 28 studies showed that taking probiotics is beneficial in AR with a reduction in Rhinoconjunctivitis Quality of Life Questionnaire (RQLQ) score and increase in Th1/Th2 cell ratios while having no effect on total IgE levels, but again not every strain is equal (142).

### 7.5 Future research directions

Several priority research directions must be pursued to advance the field. First, establishing causality between specific microbial taxa and AR onset requires large-scale longitudinal birth cohort studies (>1,000 participants with follow-up through childhood), complemented by Mendelian randomization analyses; initial genetic evidence implicating Ruminococcus gauvreauii group (OR = 1.26) and Bifidobacterium provides a starting point, but larger and geographically diverse datasets are required for validation. Second, elucidating the direct mechanistic link between the oral microbiome and AR nasal pathophysiology necessitates simultaneous multi-omics profiling of matched oral and nasal samples (microbiome–metabolome–immunome integration), with particular focus on characterizing the impact of oral pathobiont microaspiration on nasal epithelial barrier integrity and Th2 immune activation in prospective human cohorts. Third, clinical validation of individualized microbiome-targeted interventions is urgently needed: multi-center Phase III RCTs of nasal and oral probiotics using standardized endpoints (TNSS, Mini-RQLQ, microbiome reconstitution metrics) and incorporating baseline microbiome stratification to identify response-predictive biomarkers are the highest clinical research priority. Fourth, defining the optimal early-life intervention window for microbiome-based AR prevention requires randomized trials testing dietary, probiotic, and antibiotic stewardship interventions during the first 24 months of life—the immunological critical window most likely to yield durable protection against allergic sensitization.

In summary, the nasal-oral microbiome axis represents a paradigm shift in understanding AR: allergic rhinitis is no longer simply a localized Th2 immune dysregulation, but rather the systemic consequence of environment-driven microbial ecological disruption at the host–microbiome interface of the upper airway. Realizing the translational potential of this framework—from mechanistic insight to microbiome-stratified precision diagnosis and targeted therapy—will require coordinated investment in large-scale longitudinal cohorts, standardized multi-omics infrastructure, and rigorously designed intervention trials spanning the full spectrum from early-life prevention to disease-stage-specific treatment.
